# Diagnostic Accuracy with Total Adenosine Deaminase as a Biomarker for Discriminating Pleural Transudates and Exudates in a Population-Based Cohort Study

**DOI:** 10.1155/2021/6648535

**Published:** 2021-04-10

**Authors:** Bernardo Henrique Ferraz Maranhão, Cyro Teixeira da Silva Junior, Jorge Luiz Barillo, Carmem Lucia Teixeira de Castro, Joeber Bernardo Soares de Souza, Patricia Siqueira Silva, Roberto Stirbulov

**Affiliations:** ^1^Department of Specialized Medicine, Federal University of the State of Rio de Janeiro, Brazil; ^2^Department of Clinics, School of Medicine, Pleurology Teaching and Research Laboratory from Professor Mazzini Bueno Tuberculosis Research and Assistance Center, Fluminense Federal University, Niteroi, Rio de Janeiro, Brazil; ^3^General Hospital Santa Teresa, Petropolis, Rio de Janeiro, Brazil; ^4^Professor Mazzini Bueno Tuberculosis Research and Assistance Center, Fluminense Federal University, Niteroi, Rio de Janeiro, Brazil; ^5^Department of Clinics, Faculty of Medical Sciences, Santa Casa de Misericórdia de São Paulo, São Paulo, Brazil

## Abstract

**Background:**

An initial step in the evaluation of patients with pleural effusion syndrome (PES) is to determine whether the pleural fluid is a transudate or an exudate.

**Objectives:**

To investigate total adenosine deaminase (ADA) as a biomarker to classify pleural transudates and exudates.

**Methods:**

An assay of total ADA in pleural fluids (P-ADA) was observed using a commercial kit in a population-based cohort study.

**Results:**

157 pleural fluid samples were collected from untreated individuals with PES due to several causes. The cause most prevalent in transudate samples (21%, *n* = 33/157) was congestive heart failure (79%, 26/33) and that among exudate samples (71%, *n* = 124/157) was tuberculosis (28.0%, 44/124). There was no significant difference in the proportion of either sex between the transudate and exudate groups. The median values of P-ADA were significantly different (*P* < 0.0001) between both total exudates (18.4 U/L; IQR, 9.85-41.4) and exudates without pleural tuberculosis (11.0 U/L; IQR, 7.25-19.75) and transudates (6.85; IQR, 2.67-11.26). For exudates, the AUC was 0.820 (95% CI, 0.751-0.877; *P* < 0.001), with excellent discrimination. The optimum cut-off point in the ROC curve was determined as the level that provided the maximum positive likelihood ratio (PLR; 14.64; 95% CI, 2.11-101.9) and was22.0 U/L. For transudates, the AUC was 0.8245 (95% CI, 0.7470-0.9020; *P* < 0.0001). Internal validation of the AUC after 1000 resamples was evaluated with a tolerance minor than 2%. The clinical utility was equal to 92% (95% CI, 0.84 to 0.96, *P* < 0.05).

**Conclusions:**

P-ADA is a useful biomarker for distinguishing pleural exudates from transudates.

## 1. Background

Pleural effusion syndrome (PES) is determined by the interaction of dynamic phenomena that affect systemic and pulmonary circulation, lymphatic drainage, and the movements of the chest wall in many thoracic and extrathoracic diseases. Hence, the causes of PES are multiple. Therefore, based on pathophysiology, pleural effusion is traditionally classified into two types: transudate and exudate [1–3].

A diagnosis of transudate indicates that a disease has altered the systemic or pulmonary and oncotic pressures such that the balance between the formation and resorption of pleural fluid is disrupted. Unlike transudates, exudates arise from diseases or disorders in pleural membranes that increase capillary wall permeability or vascular disruption, such as infection, inflammation, infarction, and local and systemic cancer with metastasis to the pleura. Therefore, an initial step in the evaluation of patients with PES is to determine whether the pleural fluid from a thoracentesis is a transudate or an exudate [3, 4]. Light's criteria are typically employed [5]. Maranhão and Silva Junior's criterion can also be used; it has shown a diagnostic yield comparable to that of the classical Light's criteria but requires only total pleural proteins and lactate dehydrogenase in pleural fluid, without the need for serum sampling [3, 5–7]. Additional alternative criteria have been proposed in the literature [8–14]. However, ideal biomarkers in pleural fluid or serum to diagnose transudates and exudates are not yet available.

Total adenosine deaminase (ADA, adenosine aminohydrolase, enzyme code: 3.5.4.4) in pleural fluid (P-ADA) is an accurate biomarker of tuberculosis independent of human immunodeficiency virus serology status. This observation has been repeated in textbooks, reference books, and papers in recent years. While the above observation is true, the designation of total ADA as a “pleural tuberculosis biomarker” is an oversimplification [15]. ADA plays an important, active role in modulating the immune and inflammatory systems within the pleural cavity in the presence of exudative fluid [16]. The aim of the present research was to investigate total ADA as an index test or diagnostic biomarker for classifying pleural transudates and exudates.

## 2. Methods

### 2.1. Guidelines, Design, Study Population, Selection Criteria, and Sample Size

The Standards for Reporting of Diagnostic Accuracy Studies (STARD) and The Strengthening the Reporting of Observational Studies in Epidemiology (STROBE) statements were consulted to improve the quality of our study of diagnostic accuracy [17, 18]. The present research was a purely observational study with a retrospective cohort conducted from March 2015 to December 2019 at two teaching hospitals in the state of Rio de Janeiro, Brazil. The CEP CMM/HUAP Ethics Committee approved this study, which was conducted according to the guidelines of the Helsinki Declaration, under number 80/02. We obtain informed consent for this research from all patients. Pleural fluid samples were collected from continuous untreated individuals with PES due to various causes. The diagnosis of the cause of PES was confirmed by standard examinations and the use of appropriate surgical procedures [4]. Standard examinations were clinical history, physical examination, chest radiograph, and pleural ultrasound. If necessary, further image investigations were computerized tomography, positron emission tomography, and magnetic resonance imaging [4]. The classification of a transudate or exudate was established by clinical judgment and assays of total protein and total lactate dehydrogenase in pleural fluids [3, 6]. The causal diagnosis of PES was confirmed after one or two thoracentesis procedures with standard laboratory evaluation of the pleural fluid with appearance, odor, cytological analysis, and constituent assays (total proteins, lactate dehydrogenase, glucose, pH, amylase, triglycerides and cholesterol, and natriuretic peptides), biomarkers for connective tissue diseases, bacterial pleural infections, and tuberculous pleurisy. If the PES persisted without a causal diagnosis, the patient was forwarded for video-assisted thoracoscopic surgery with an indication of pleural biopsy for histopathological diagnosis [4]. Exclusion criteria included contraindications or refusal for surgical procedures, the use of immunosuppressive drugs, hemolysis in pleural liquids, renal failure, human immunodeficiency virus infection, and PES of unknown cause. In addition, patients with serum levels of bilirubin greater than 65 mg/dL, lipid greater than 1500 mg/dL, and rheumatoid factor content greater than 1500 IU/M were excluded because these biochemical factors interfere with P-ADA [19]. According to the exclusion and inclusion criteria and the calculated minimum sample size, one hundred fifty-seven pleural fluids from 157 patients with PES with various confirmed diagnoses were selected for this study [17].

### 2.2. ADA Assay

Pleural fluid ADA activity was evaluated using the Diazyme Assay (Diazyme Laboratories, San Diego (CA), United States). ADA irreversibly catalyzes the conversion of adenosine (or deoxyadenosine) to inosine (or deoxyinosine) and ammonia. The classical method of Giusti and Galanti is not easily automated because the ammonia is measured with Berthelot's reaction. Hopkinson et al. (1969) described an enzyme assay for ADA determination based on nucleoside phosphorylase (NP) and xanthine oxidase (XOD). The quantification of ADA was based on the measurement of uric acid after enzymatic reactions with NP, XOD, inosine, and other reagents. Briefly, the Diazyme ADA Assay uses a kinetic method. It is based on the enzymatic deamination of adenosine to inosine which is converted to hypoxanthine by NP. Hypoxanthine is then converted to uric acid and hydrogen peroxide by XO. One unit of total ADA was defined as the amount of the enzyme that generates one micromol of inosine per min at 37°C from the substrate adenosine [19]. P-ADA assay was performed independently of the proven diagnosis of the patient in a hospital with the same health professional and conducted according to the manufacturer's guidelines.

### 2.3. Statistical Model Development

All quantitative and qualitative data from our patient charts were compiled in MS-Excel 2010 version 2010. Descriptive and inferential statistical analyses were performed using GraphPad (GraphPad Software, Inc., version 6.0, La Jolla, CA, USA). Laboratory data were analyzed with univariate statistical tests. A *P* value less than 0.05 determined from a two-tailed test was considered indicative of statistically significance, leading us to reject the null hypothesis with a 5% probability of a type I error. The Shapiro and Wilk test was used to evaluate the data distributions. Quantitative variables that were normally distributed were expressed as means and standard deviations, and those with nonnormal distributions were expressed in terms of their medians and interquartile range (IQR, 25th and 75th percentiles). Proportions were used to express qualitative or categorical variables. To compare proportions, a Chi-square test was employed. The Mann–Whitney *U* nonparametric test was used to compare the median of pleural total ADA level between transudates and exudates when the data were not normally distributed. The receiver operating characteristic curve (ROC curve) method with logistic regression was used to select the best P-ADA cut-off value to classify pleural exudates. The nonparametric (empirical) method by DeLong et al. (1988) was used for computing the ROC curve according to our analysis of the normality of P-ADA data distributions. This method was applied because it does not make the strong normality assumptions that the binormal method makes. The criterion used to select an “optimal” cut-off point was the maximum positive likelihood ratio (PLR) for exudates. Thus, a cut-off with high specificity and a low rate of false positives and false negatives for exudates was obtained [20–26].

### 2.4. Performance Measures: Diagnostic Accuracy, Discrimination, Calibration by Internal Validation, and Clinical Utility

Diagnostic accuracy measures the ability of an index test to detect a condition when it is present and detect the absence of a condition when it is absent. Sensitivity, specificity, predictive values, likelihood ratios, and diagnostic odds ratio are measures of diagnostic accuracy. The diagnostic accuracy of P-ADA for exudates and transudates was quantified by positive likelihood ratio (PLR). According to a robust scale, biomarkers with a PLR greater than 10 or an NLR less than 0.1 have the potential to alter clinical decisions, and its results are conclusive. Diagnostic biomarkers with PLRs between 5 and 10 or 0.1 and 0.2 often provide useful additional information, whereas biomarkers with PLRs ranging from 0.33 to 3 rarely alter clinical decisions [20–26].

Discrimination was measured by the *c*-statistic (which is equal to the area under the ROC curve space, AUC) and 95% confidence intervals. A general classification scheme of the discrimination accuracy of a biomarker by AUC was proposed by Hosmer and Lemeshowand adopted by other authors and is as follows [20–26]: excellent discrimination (0.90-1.0), very good discrimination (0.80-0.90), good/acceptable discrimination (0.70-0.80), sufficient (0.60-0.70), poor (0.50-0.60), and biomarker not useful (0.00-0.50).

Calibration, or reliability, or validation, or goodness-of-fit, or overall fit approaches are methods for validating the statistical results. Calibration can be defined as the agreement between observed outcomes and predictions. The Bootstrap method of internal validation was applied for calibration. This classical method evaluated the potential overfitting of the AUC in this accuracy model. The aim of internal validation is to quantify the model's predictive performance in either resampled participant data of the development data set. Here, a bootstrap confidence interval (1000 resamples; a random number seed of 978) was employed to generate a 95% CI of AUC. A tolerance 0.02 with 1000 replications was considered appropriate [27].

The clinical utility of a biomarker depends on its diagnostic accuracy, the pretest probability of disease, and the clinical consequences of the test results. Clinical utility is a qualitative metric. A clinical utility index (CUI) for diagnostic tests was employed to evaluate this accuracy model [28–30]. The performance of the P-ADA biomarker was evaluated with MedCalc for Windows, version 19.3 (MedCalc Software, Ostend, Belgium).

## 3. Results

### 3.1. Sociodemographic and Laboratory Characteristics

Table 1 summarizes the prevalence of the causes of PES among the 157 patients in the study site and timeframe. Exudates were more prevalent (79%) than transudates (21%). The more common cause of transudate was congestive heart failure (79%, 26/33). Tuberculosis was in exudates (28.0%, 44/124).

The demographic characteristics and P-ADA levels of the 157 patients are presented in Table 2. The median values of the variable P-ADA were significantly different (*P* < 0.0001) between both exudates (18.4 UI/L; IQR, 9.85-41.4) and exudates without pleural tuberculosis (11.0 UI/L; IQR, 7.25-19.75) and transudates (6.85; IQR, 2.67-11.26). There was no significant difference in the proportion of either sex between the transudate and exudate groups, as determined by Chi-square test (*P* values: males, 0.9415; females, 0.9416).

### 3.2. Performance Measures

**Figure 1:**
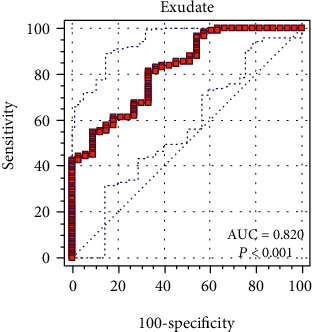
ROC curve of pleural fluid values and 95% CIs of adenosine deaminase for the diagnosis of exudates. The optimum cut-off point in ROC curve space was determined as the level that provided the maximum positive likelihood and was 22.0 U/L. AUC = 0.820 (95% CI, 0.751-0.877; *Z* = 8.157; SE, 0.0393; *P* < 0.001). Abbreviations: CIs: confidence intervals; AUC: area under the ROC curve; SE: standard error.

Figure 1 shows the ROC curve of P-ADA values for the diagnosis of exudates. For exudates, the AUC was 0.820 (95% CI, 0.751-0.877; *Z* = 8.157; SE, 0.0393), with a highly significant *P* value (*P* < 0.001). The optimum cut-off point in ROC curve space was identified as the level that provides the highest positive likelihood and was 22.0 U/L. For transudates, the AUC was 0.8245 (95% CI, 0.7470-0.9020; SE, 0.03955), with a highly significant *P* value (*P* < 0.0001). The optimum cut-off point in ROC curve space was determined to be 22.0 U/L.

The results of the ROC curve analysis of the accuracy of the P-ADA when employing a statistically appropriate cut-off point are shown in Table 3.

Table 4 shows the internal validation by bootstrapping of the 95% confidence interval of the area under the ROC curve after 1000 resamples for the classification of pleural exudate and transudate with total adenosine deaminase.

## 4. Discussion

In a diagnostic accuracy study, the results from an index test are compared with the results obtained with the reference standard on the same subjects. However, before a biomarker to be recommended for clinical practice, it should be evaluated for analytical, clinical, and internal validity. Moreover, external validation and clinical utility are desirable [28, 31, 32].

Analytical validity is performed under rigorous laboratory control and encompasses tests of sensitivity, specificity, trueness, bias (precision, repeatability), reproducibility, detection and quantification limits, linearity, range, and robustness [31]. The correlation of Diazyme ADA commercial kit with the classical Giusti method was 0.93 according to Delacour et al. [19].

Clinical validity is evaluated in diagnostic accuracy studies and refers to a test's accuracy in discriminating between subjects with or without a disease. A difficulty encountered in accuracy studies is deciding how to estimate an optimal threshold for a biomarker. An ideal cut-off point should be determined for each region and each disease evaluation. It is not a universal number [20–26].

Bossuyt et al. [28] defined the clinical utility of a biomarker or diagnostic test as “the degree to which actual use of the corresponding test in healthcare is associated with changing health outcomes, such as preventing death and restoring or maintaining health.” In other words, clinical utility refers to whether the biomarker or test can be used for a specific diagnosis or whether it is suitable for diagnostic purposes. Randomized controlled trials (RCTs) are the best context for evaluating the clinical utility of a biomarker [28, 31]. However, RCTs are rarely conducted, due to economic and ethical reasons. Furthermore, an RCT is not always necessary to evaluate a diagnostic test.

Asberg et al. [29] proposed a new index of clinical utility (CUI). We calculated a CUI for this accuracy model of 92% using the formula recommended by authors with the treatment threshold of Pauker and Kassirer [30]. The 95% confidence interval extended from 0.8479 to 0.9610 (*P* < 0.05).

Tuberculosis is the most prevalent cause of exudative pleural effusions in Brazil according to Table 1 and another paper recently published by our group [33]. Furthermore, the sex ratio and median patient age in our study were similar to those in a study of a Brazilian sample population from a metropolitan region in the state of Rio de Janeiro, as shown in Table 2 [33]. Female sex was more prevalent than male sex in exudates and transudates, although the differences were not significant, as determined by Chi-square test (Table 2). There was a significant difference between the age of patients with exudate and transudate in Table 2 (*P* < 0.0001, Mann–Whitney test). Perhaps, the causes of transudates mentioned in Table 1 were important for the results.

The median values of total P-ADA activity in the pleural fluid of patients with exudates and transudates were 18.4 U/L and 6.85 U/L, respectively, with a highly significant *P* value (*P* = 0.0001) as determined by Mann–Whitney test (Table 2).

Although P-ADA has been identified as the most accurate biomarker available for pleural tuberculosis in clinical practice, we found that total P-ADA discriminated between transudates and exudates even when the tuberculosis samples were excluded (*U* = 665.5, *P* < 0.0001), as shown in Table 2.

ROC curve space can be used to identify optimal cut-off values for a given biomarker in discriminating between patient states, traditionally referred to as diseased and no diseased [22]. The ROC curve for exudates is shown in Figure 1. The ROC curve for transudates is not shown. The AUC calculated for exudates was 0.820 (95% CI, 0.751-0.877; *P* < 0.001) and that calculated for transudates was 0.8245 (95% CI, 0.7470-0.9020; *P* < 0.0001). According to the classification of Hosmer and Lemeshow, an AUC equal of 0.820 is indicative of excellent discrimination (0.80-0.90). In practice, AUCs greater than 0.90 are uncommon [26]. A 95% CI from AUC of 0.736 and 0.889 was obtained after 1000 resamples, and a tolerance less than 0.02 was calculated (Table 4).

A few previous have investigated the utility of P-ADA for the diagnosis of transudates and exudates. Similar to our work, they all obtained AUCs indicating excellent or very good discrimination [34–36]. The AUC space reflects the predictive performance of a biomarker. A large advantage of the ROC curve method is that the AUC space is agnostic. However, the choice of a cut-off threshold should not be, as it is dependent on several statistical and clinical criteria [20, 22, 37].

The likelihood ratio can serve as a powerful measure of the diagnostic accuracy of a biomarker [3, 26]. The PLR is the ratio of the probability of a positive test result given the presence of the disease to the probability of a positive test result given the absence of the disease. The choice of our cut-off point in ROC curve space according to the maximum positive likelihood of P-ADA for the detection of exudates demanded high specificity even at the expense of lower sensitivity (Table 3). A maximum PLR of 14.64 for exudates in our study corresponded to a cut-off point of 22.0 UI/L of P-ADA and has the potential to alter clinical decisions [22, 25]. As evident from the transudate results in Table 3, with a PLR of 1.7 and a low specificity of 42%, a P-ADA level of 22.0 U/L is very useful for classifying exudates but not transudates.

The diagnostic accuracy of the traditional approach (Light's criteria) and ADA criterium in the present research were compared. A paper from our group was used [6]. For exudates, Light's criteria were 94%. In Table 3, predictive ADA criterium was 54%. However, the PLR from traditional criterium was 3.17. In Table 3, the PLR was 14.64 with ADA criterium. Currently, the likelihood ratio is the best measure of the diagnostic accuracy of a biomarker [3, 26].

In Bhopal, India, without using the ROC curve method, Jadhav and Bardapurkar also selected a cut-off point of 22.0 U/L of P-ADA for the diagnosis of exudates. A PLR for accuracy was not calculated by the authors [35].

The present research has limitations. First, the results must be interpreted in consideration of the several criteria proposed in the literature for selecting an optimal cut-off point of a biomarker in ROC curve space. Second, our cut-off value for diagnosis of pleural exudate and transudate must be applied to a sample population similar in demographic characteristics, prevalence of PES causes, and P-ADA assay method. Third, no universal cut-off value exists for a diagnostic biomarker. Despite these limitations, we are optimistic about the future perspectives of our study. Total P-ADA is an accurate biomarker of pleural tuberculosis. Thus, the same biomarker can be used for two different purposes, although with different cut-off points: diagnosis of pleural tuberculosis and classification of transudates and exudates.

## 5. Conclusions

The results demonstrate that P-ADA is a useful biomarker to differentiate exudates from transudates. However, several other criteria mentioned in the literature also must be considered for clinical decision-making.

## Figures and Tables

**Table 1:** Prevalence of the causes of pleural effusion syndrome evaluated in 157 patients from March 2015 to December 2019.

Cause	No. of patients	Prevalence (%)
Transudates^∗^	33	21.0
Total exudates	124	79.0
Tuberculosis	44	28.0
Adenocarcinoma	37	23.0
Simple parapneumonic effusions	15	9.0
CPPE and empyema	8	5.0
Lymphoma	7	5.0
Squamous cell carcinoma	7	5.0
Other exudates^‡^	6	4.0

Abbreviations: CPPE: complicated parapneumonic effusions. ^∗^Transudates: congestive heart failure (*n* = 26), chronic renal failure (*n* = 3), cirrhosis of the liver with ascites (*n* = 3), and serum low total protein levels (*n* = 1). ^‡^Other exudates: pseudo-Meigs' syndrome (*n* = 1), Dressler's syndrome (*n* = 3), chylothorax (*n* = 1), and leukemia (*n* = 1).

**Table 2:** Demographic characteristics and pleural adenosine deaminase levels in 157 cases of exudates and transudates.

Variable	Exudate (*n* = 124)	Transudate (*n* = 33)	*P* value
Age, years, median (IQR)	58. (41.5-73.5)	76.0 (63.0-86.25)	0.0001
Male, *n* (%)	58 (47.0)	16 (48.0)	
Female, *n* (%)	66 (53.0)	17 (52.0)	
P-ADA, U/L, median (IQR)	18.4 (9.25-41.4)	6.85 (2.67-11.26)	0.0001
P-ADA, U/L, without pleural TB, median (IQR)	11.0 (7.25-19.75)	6.85 (2.67-11.26)	0.0001

^∗^Shapiro-Wilk normality test: *P* < 0.05; Abbreviations: IQR: interquartile range of 75%-25%; P-ADA: pleural adenosine deaminase; TB: tuberculosis.

**Table 3:** Measures of diagnostic accuracy of adenosine deaminase assay following selection of the best cut-off point for pleural exudates and transudates according to the maximum positive likelihood ratio in the ROC curve space.

Diagnostic parameter, % (95% CI)	Exudate (*n* = 124)	Transudate (*n* = 33)
Best cut-off (U/L)	≥22.0	<22.0
Sensitivity	44.35 (35.4-53.5)	96.88 (83.78-99.92)
Specificity	96.98 (83.0-99.0)	42.92 (34.01-52.29)
Positive predictive value	98.11 (88.0-99.0)	29.83 (26.48-33.42)
Negative predictive value	31.0 (27.0-34.0)	98.27 (89.06-99.75)
Positive likelihood ratio	14.64 (2.11-101.9)	1.7 (1.44-2.01)
Negative likelihood ratio	0.57 (0.50-0.70)	0.07 (0.01-0.49)
Diagnostic odds radio	23.26 (3.08-176.75)	24.28 (144.0-4.10)
Predictive accuracy	54.25 (46.0-62.0)	53.77 (45.57-61.83)
Disease prevalence	79.0	21.0

None of the 95% confidence intervals (CIs) overlap 1 (percentages) or 0 (absolute values), indicating the differences are significant at *P* < 0.05.

**Table 4:** Calibration with internal validation by bootstrapping of the 95% confidence interval of the area under the ROC curve after 1000 resamples for the classification of pleural exudate and transudate with total adenosine deaminase.

Development AUC		Internal validation for optimism-correct AUC (95% CI)	Variation	Chi-squared test
95% CI	Observed	Expected	%	*P* value
Minimum	0.751	0.736	-1.99	0.092 (*P* = 0.7612)
Maximum	0.877	0.883	+ 0.68	0.027 (*P* = 0.8703)

A tolerance 2% with 1000 replications was considered appropriate. Abbreviations: 95% CI: 95*%* confidence Interval; AUC: area under the ROC curve.

## Data Availability

The Tables and Figure data used to support the findings of this study are included within the article.

## Abbreviations

ADA: Total adenosine deaminase
AUC: Area under the ROC curve
CI: Confidence interval
CUI: Index of clinical utility
IQR: Interquartile range
NLR: Negative likelihood ratio
P-ADA: Total ADA in pleural fluids
PES: Pleural effusion syndrome
PLR: Positive likelihood ratio
RCTs: Randomized controlled trials
ROC curve: Receiver operating characteristic curve
STARD: The Standards for Reporting of Diagnostic Accuracy Studies.
